# Anti-Inflammatory Effect of Columbianadin against D-Galactose-Induced Liver Injury In Vivo via the JAK2/STAT3 and JAK2/p38/NF-κB Pathways

**DOI:** 10.3390/ph17030378

**Published:** 2024-03-15

**Authors:** Zhe Ma, Lin Peng, Yaoyao Sheng, Wenhui Chu, Yongqian Fu

**Affiliations:** 1 School of Life Science, Taizhou University, 1139 Shifu Avenue, Taizhou 318000, China; tzcmz@tzc.edu.cn (Z.M.); leogard2005@163.com (L.P.); shengyaoyao0902@163.com (Y.S.); 2 Taizhou Key Laboratory of Biomass Functional Materials Development and Application, Taizhou University, Taizhou 318000, China

**Keywords:** anti-inflammation, columbianadin, coumarins, D-galactose-induced liver injury, oxidative stress

## Abstract

*Angelicae pubescentis* radix (APR) has been traditionally used for thousands of years in China to treat rheumatoid arthritis (RA), an autoimmune disorder. As the main active coumarin of APR, columbianadin (CBN) exhibits a significant anti-inflammatory effect in vitro. However, the anti-inflammatory activity and underlying mechanism of CBN in vivo remain unclear. This work aimed to elucidate the anti-inflammatory activity of CBN in vivo and its related signaling pathways in a D-Gal-induced liver injury mouse model. Analysis of biochemical indices (ALT and AST) and pro-inflammatory cytokines (IL-1β and IL-6) in serum indicated that CBN significantly ameliorated D-Gal-induced liver injury. CBN treatment also significantly increased the activities of antioxidant enzymes (SOD, CAT, GPx), and decreased the levels of pro-inflammatory cytokines (TNF-α, IL-1β and IL-6) in liver tissue. Liver histology revealed that CBN treatment reduced hepatic inflammation. Western blot analysis indicated that CBN down-regulates the expression of phosphorylated JAK2, STAT3, MAPK, and NF-κB in the related signaling pathways. These findings support the traditional use of APR as a remedy for the immune system, and indicate that the JAK2/STAT3 and JAK2/p38/NF-κB signaling pathways may be important mechanisms for the anti-inflammatory activity of CBN in vivo.

## 1. Introduction

*Angelicae pubescentis* radix (APR), the root of *Angelica pubescens* (Apiaceae) Maxim. f. *biserrata* Shan et Yuan, is a traditional Chinese medicine mainly grown and distributed in the Sichuan and Hubei provinces in China. APR has been an important constituent of Chinese medicine prescriptions for treating rheumatoid arthritis (RA) for thousands of years and exhibits an analgesic effect [1]. RA is a chronic autoimmune disorder characterized by the disruption of the innate and adaptive immune system, which promotes inflammation of the joints [2]. The medical use of APR is closely associated with the active compounds in APR. The major bioactive components in APR are coumarins and volatile oils [3]. Approximately 70 coumarins, including columbianadin (CBN), osthole (OE), columbianetin (CBT), columbianetin acetate (CBA), isoimperatorin, and bergapten, have been identified and isolated from APR [4]. Among these components of coumarins and volatile oils, CBN is identified as a standard ingredient of APR and its level in APR is required to be no less than 0.08% according to the *Chinese Pharmacopoeia* (version 2020). Therefore, CBN may be the main active component in APR and seems to act as anti-inflammatory agent in medical applications of APR.

Previous studies have shown that CBN exhibits calcium-channel-blocking activity [5], inhibiting platelet aggregation [6], cytotoxic activity against different human carcinoma cell lines [7], and anti-inflammatory activity [8,9]. Following studies further proved the anti-inflammatory effect of CBN. APR root extracts containing CBN have been reported to exhibit anti-inflammatory activity in mice and rats in previous studies [9,10]. Furthermore, CBN exhibited an immunosuppressive effect in lipopolysaccharide (LPS)-stimulated THP-1 cells [11]. Another previous study [12] also reported that CBN inhibited cell maturation via its immunosuppressive effects in dendritic cells (DCs). In addition, the biotransformation products of CBN also exhibited anti-inflammatory activities. In a rat hepatic microsomal model in vitro, 13 biotransformation products including CBT were identified and further exhibited great anti-inflammatory effects by reducing NO production in LPS-stimulated murine RAW264.7 macrophages in vitro [13]. It is noteworthy that CBT exhibits anti-inflammatory activity in various cell lines, including human HT-29 colon carcinoma cells [8] and human peripheral blood mononuclear cells [14]. Therefore, CBN and its biotransformation products after absorption have great potential value to be developed as pharmaceutical agents with anti-inflammatory properties.

Despite this evidence of the immunosuppressive effect of CBN in vitro, studies focusing on its anti-inflammatory effect in vivo and the related mechanisms are scarce. A previous study showed that the transportation of CBN into the blood mainly occurs in hepatic tissue [15]. Therefore, CBN may exert hepatoprotective effects against liver injury and diseases via its immunosuppressive effect. In this work, the immunosuppressive and hepatoprotective effects of CBN were investigated in a D-Gal-induced liver injury mouse model. Meanwhile, the underlying mechanisms of the hepatoprotective and immunosuppressive effects of CBN were also elucidated. This work validates a new anti-inflammatory agent identified from this traditional medicinal herb and demonstrates its potential value.

## 2. Results

### 2.1. Influence on Body Weight and Liver Indices

ICR mice injected with D-Gal were fed with CBN at various doses to investigate its anti-inflammatory effect, and the results are presented in Figure 6. The mice in the six groups showed an increase in their final body weight (BW) compared with their initial BW during the 8-week experimental period (Table 1). The weight loss was clarified by comparing the final BWs in the control and model groups (*p* < 0.05). The positive group (VE) and the CBN groups (200–800 mg/kg BW) exhibited a slower decrease in BW compared with the model group. The results in Table 1 display the liver indices after 8 weeks of feeding. The liver index in the model group was significantly lower than in the control group (*p* < 0.05), and treatment with CBN and VE slowed down this trend. Moreover, the high dose of CBN (800 mg/kg BW) obtained a similar liver index to the VE group. Our findings reveal that CBN has a protective effect against D-gal-induced reductions in BW and liver index.

### 2.2. Influence on Serum Biochemicals and Inflammatory Cytokines

**Figure 1:**
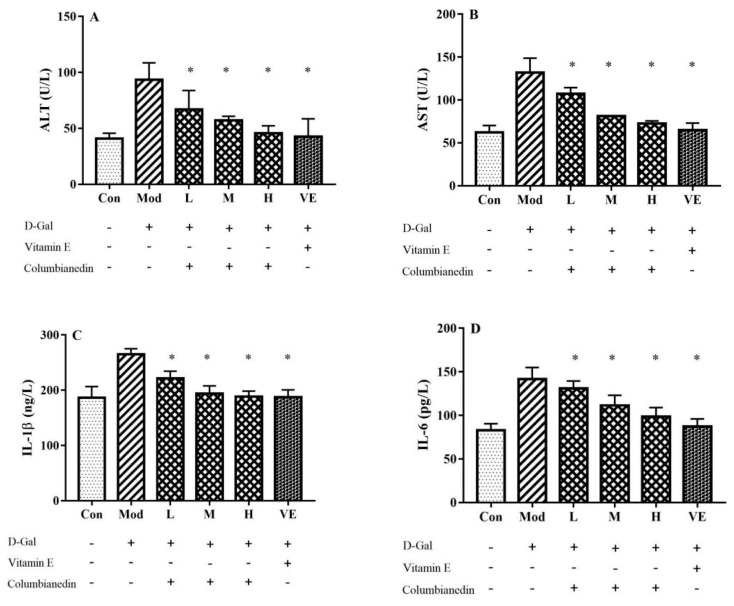
Effect of various doses of CBN on serum biochemical indicators in a D-Gal-induced aging mouse model. Increased serum levels or activities of (**A**) ALT; (**B**) AST; (**C**) IL-1β; (**D**) IL-6 caused by D-Gal were effectively reduced through treatment with various doses of CBN. Con: control group; Mod: model group (D-Gal); L: D-Gal + 200 mg/kg/BW CBN; M: D-Gal + 400 mg/kg/BW CBN; H: D-Gal + 800 mg/kg/BW CBN; VE: positive group. * *p* < 0.05 vs. model group.

The serum activity levels of ALT, AST, IL-1, and IL-6 were determined in this study (Figure 1). The serum ALT and AST levels in the model group were approximately two-times higher than those in the control group (*p* < 0.05), which indicated severe liver injury. CBN treatment with doses of 200–800 mg/kg/BW and the positive drug VE significantly reduced the in-serum activities of ALT and AST (*p* < 0.05). Similarly, the serum levels of IL-1β and IL-6 in the high-dose CBN group (800 mg/kg/BW) were significantly decreased by 28.8% (*p* < 0.05) and 30.5% (*p* < 0.05), respectively, compared with the model group, respectively. These results suggest that CBN normalizes serum ALT and AST activities, and alleviates the inflammatory response in the serum.

### 2.3. Influence on D-Gal-Induced Oxidative Stress in Liver Tissue

**Figure 2:**
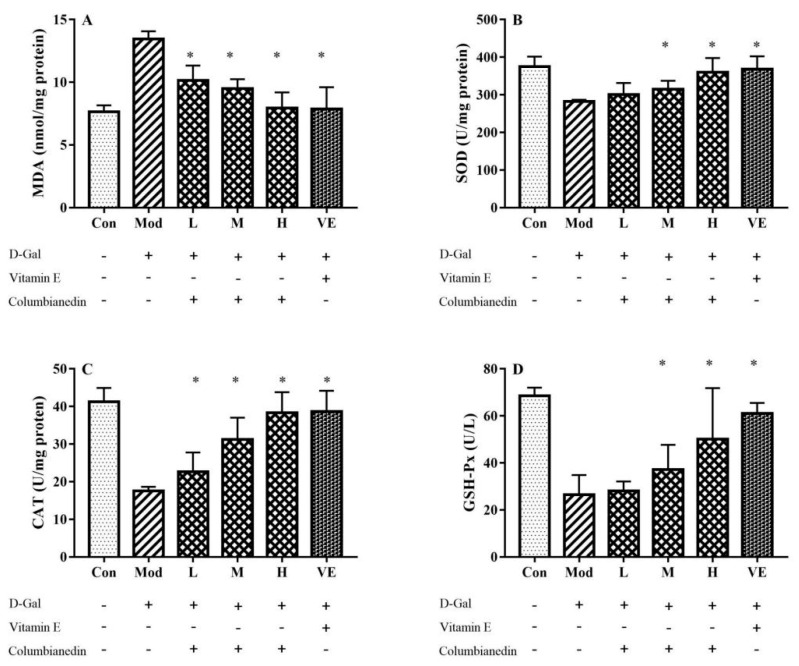
Effect of various doses of CBN on liver oxidative stress index in a D-Gal-induced aging mouse model. After treating with various doses of CBN, abnormal hepatic activities or levels of (**A**) MDA; (**B**) SOD; (**C**) CAT; (**D**) GPx were effectively restored compared to the model group. Con: control group; Mod: model group (D-Gal); L: D-Gal + 200 mg/kg/BW CBN; M: D-Gal + 400 mg/kg/BW CBN; H: D-Gal + 800 mg/kg/BW CBN; VE: positive group. * *p* < 0.05 vs. model group.

In the model group, D-Gal feeding significantly increased the MDA level and decreased the activities of SOD, CAT, and GPx in hepatic tissues compared with the control group (Figure 2). The CBN treatment effectively reduced the MDA level in the liver tissue. The maximal reduction (approximately 41%) was achieved with the CBN dose of 800 mg/kg/BW, similar to the VE group (Figure 2A). The antioxidant enzyme activities were restored by CBN in a dose-dependent manner (Figure 2B–D). The liver tissue activities of SOD, CAT, and GPx in the CBN groups were all significantly higher than those in the model group (*p* < 0.05), and activities were recovered to levels similar to the control group levels. Specifically, the activities of SOD, CAT, and GPx with the CBN dose of 800 mg/kg/BW were significantly increased 27.3%, 118%, and 106%, respectively, comparing to the model group. These results indicate that CBN has a protective effect against D-Gal-induced oxidative stress in the liver.

### 2.4. Influence on Inflammatory Cytokines in Liver Tissue

**Figure 3:**
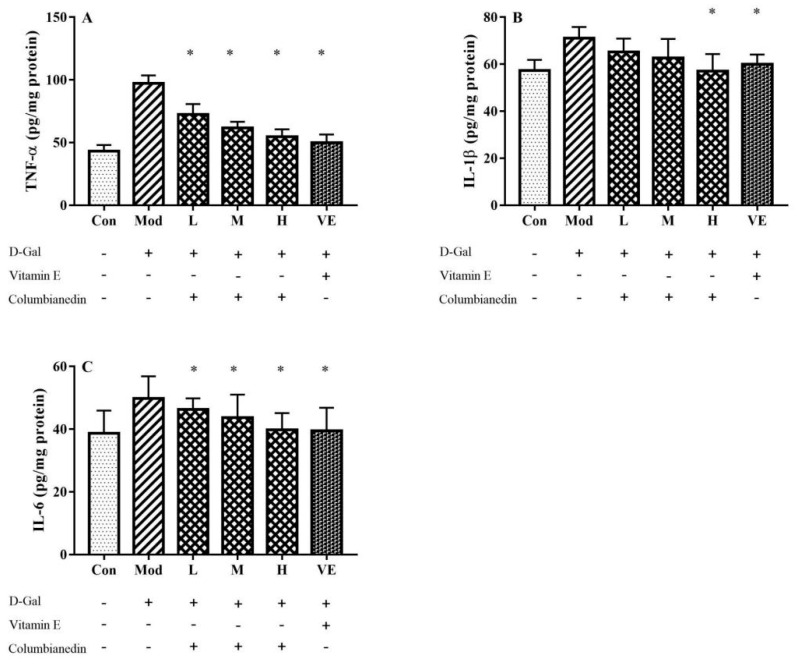
Effects of various doses of CBN on liver inflammatory cytokines in a D-Gal-induced aging mouse model. Hepatic levels of (**A**) TNF-α; (**B**) IL-1β; (**C**) IL-6 were effectively reduced by treating the mice with CBN in the experimental groups, compared to the model group. Con: control group; Mod: model group (D-Gal); L: D-Gal + 200 mg/kg/BW CBN; M: D-Gal + 400 mg/kg/BW CBN; H: D-Gal + 800 mg/kg/BW CBN; VE: positive group. * *p* < 0.05 vs. model group.

The effects of CBN on the expressions of TNF-α, IL-1β and IL-6 in liver tissue were determined, and the results are shown in Figure 3. Compared with the control group, the levels of TNF-α, IL-1β, and IL-6 were significantly increased in the model group (*p* < 0.05), indicating the D-Gal-induced inflammation reaction. CBN treatment effectively ameliorated the abnormal expression of pro-inflammatory cytokines in D-Gal-induced injury liver tissue in a dose-dependent manner, which was similar to the results of VE treatment. Compared to the control group, pro-inflammatory cytokines in the model group were increased by 126% (TNF-α), 24% (IL-1β), and 29% (IL-6). After treating with the CBN dose of 800 mg/kg/BW, levels of TNF-α, IL-1β, and IL-6 in the liver tissue were decreased by 44%, 20.4%, and 21.8%, respectively. Based on this study, it can be concluded that CBN alleviates D-Gal-induced liver injury and improves liver function by reducing the expression of pro-inflammatory cytokines in liver tissue.

### 2.5. Influence on Liver Histology

**Figure 4:**
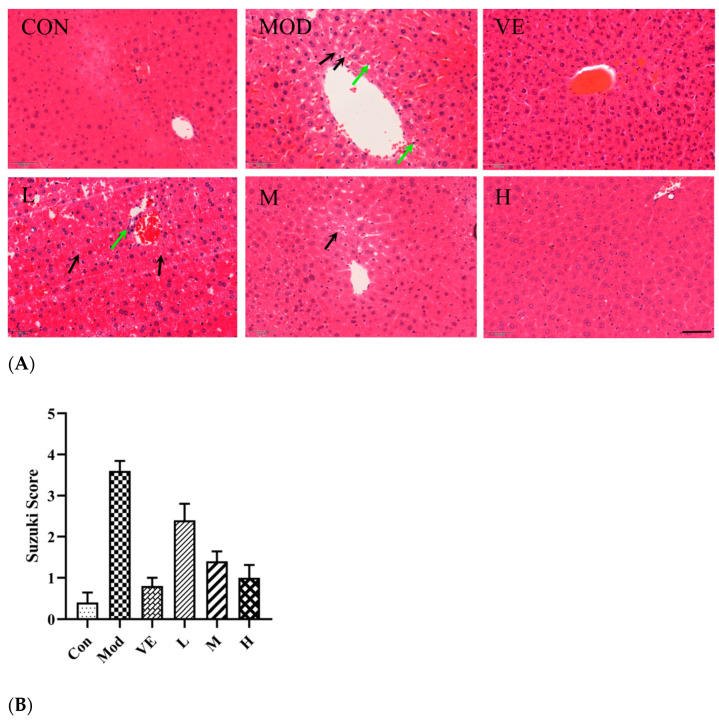
CBN treatment alleviated D-Gal-induced liver injury in the mouse model and reduced edema (black arrow) and inflammatory cell infiltration (green arrow) in hematoxylin and eosin (H&E) staining analysis (**A**). Suzuki Scores analysis (**B**) was carried out to measure the amelioration degree of CBN treatment. Con: control group; Mod: model group (D-Gal); L: D-Gal + 200 mg/kg/BW CBN; M: D-Gal + 400 mg/kg/BW CBN; H: D-Gal + 800 mg/kg/BW CBN; VE: positive group. Scale bar: 50 μm; magnification power: ×400.

As shown in Figure 4, the liver lobule structure in the control group was clear and complete with no edema or inflammatory cell infiltration. After feeding with D-Gal for 8 weeks, hepatocytes around the central vein in the liver tissue of the model group were enlarged and exhibited apparent inflammatory cell infiltration (Figure 4). The results of the CBN treatment (200–800 mg/kg/BW) exhibited a disordered arrangement, but the edema and inflammatory cell infiltration were reduced compared with the model group. The hepatic lobule structure in the CBN groups almost returned to normal, which was similar to the VE group (Figure 4A). The hepatoprotective effects of CBN treatment in the liver histology were consistent with the Suzuki System Scores (Figure 4B). This result indicates that CBN can significantly reduce liver cell apoptosis and ameliorate tissue damage in a dose-dependent manner as well as liver injury induced by D-Gal.

### 2.6. Influence on the Expression of Proteins in JAK2/STAT3 and JAK2/p38/NF-κB Signaling Pathways

**Figure 5:**
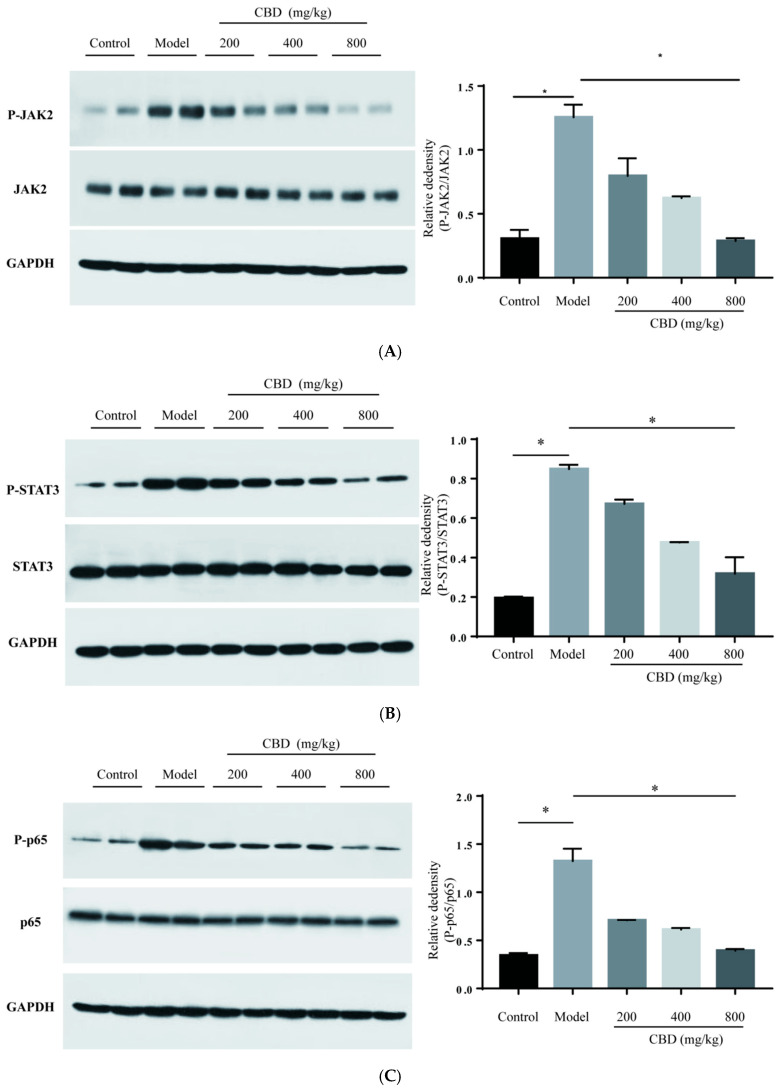

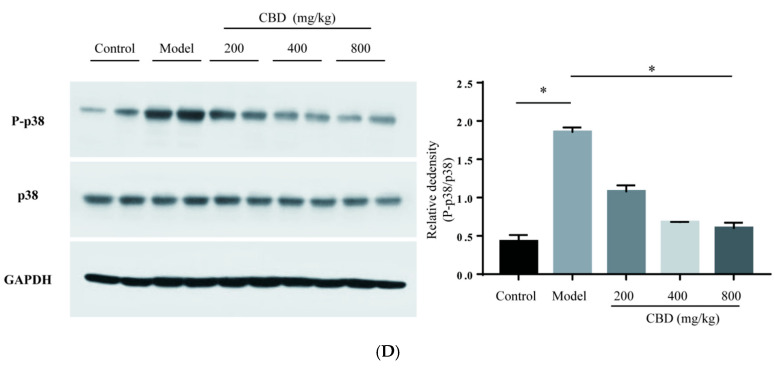
Effect of CBN on JAK2, STAT3, NF-κB, and MAPK signaling pathways in liver tissue. The protein expression levels of (**A**) P-JAK2 and JAK2; (**B**) P-STAT3 and STAT3; (**C**) P-p65 and p-65; and (**D**) P-p38 and p-38. CBN affected the phosphorylation of key proteins in JAK2, STAT3, and other signaling pathways. * *p* < 0.05 vs. model group.

In this study, the increased level of phosphorylated JAK2 (P-JAK2) and P-STAT3 in the model group was reduced after treatment with CBN, and this reducing trend of P-JAK2 and P-STAT3 remained consistent with the increasing CBN doses (Figure 5A,B). Specifically, phosphorylation of JAK2 and STAT3 with the CBN dose of 800 mg/kg/BW was decreased by 3.3 and 1.6 times compared to the model group. Some pro-inflammatory cytokines, such as IL-6 and TNF-α, act as autocrine feedback signals and can also activate NF-κB phosphorylation. Our results show that the expression level of IL-6 was enhanced in the model group, and these pro-inflammatory cytokines may activate NF-κB phosphorylation (Figure 2C). The NF-κB and MAPK pathways are reported to be involved in the inflammation process, and have become the target of anti-inflammatory chemical screening and identification. In this study, the expression levels of phosphorylated p65 (P-p65, a subunit of the NF-κB dimer) and phosphorylated p38 (P-p38, belonging to the MAPK family) all decreased after CBN treatment (Figure 5C,D). Specifically, phosphorylation of p65 and p38 with the CBN dose of 800 mg/kg/BW was decreased by 2.3 and 2.1 times compared to the model group. These results indicate that the activation of the JAK2/STAT3 and JAK2/NF-κB/MAPK signaling pathways caused by D-gal-induced liver injury in a mouse model can be attenuated through CBN treatment.

## 3. Discussion

According to its traditional use and the *Chinese Pharmacopoeia*, APR has the clinical effects of dispelling wind to eliminate dampness, removing arthralgia, and stopping pain [1]. Coumarins in APR act as the main bioactive components and exhibit anti-inflammatory activities, which are closely associated with the treatment of RA in traditional medicine. In previous studies, CBN was shown to exhibit great anti-inflammatory activity and affect related signaling pathways in various cell lines, including TNF-α-induced dendritic cells, LPS-induced RAW264.7 macrophages, and LPS-induced THP-1 cells [11,12,13]. However, few studies have focused on the underlying mechanisms of CBN in vivo. Zhang et al. reported that biotransformation of CBN occurred in a rat hepatic microsomal model and 13 biotransformation products also exhibited significant anti-inflammatory activity [13]. Based on these studies, CBN could exert hepatoprotective effects against liver injury via anti-inflammation and related signaling pathways. In this study, the anti-inflammatory activity of CBN was investigated in a D-Gal-induced liver injury mouse model in order to gain a better understanding of the underlying mechanism.

During the 8-week experimental period, the final body weights (BWs) of the mice in the six groups increased compared with their initial BWs (Table 1). However, a weight loss was shown by comparing the final BWs between the control and model groups (*p* < 0.05). The positive group (vitamin E) and those treated with various doses of CBN (200–800 mg/kg BW) demonstrated a relatively slower decrease in BW compared with the model group. Table 1 shows the liver indices after the 8-week feeding period. The liver index in the model group was significantly lower than in the control group (*p* < 0.05), and the treatments with CBN and VE slowed the decreasing trend. Furthermore, the high dose of CBN (800 mg/kg BW) showed similar liver indices to the VE group. Xu et al. [16] reported that aging mice fed with D-galactose showed reductions in BW and organ indices compared with control group mice. Our results indicate that CBN protects against D-Gal-induced BW and liver index losses.

Balance between the oxidation and antioxidation processes in vivo is necessary to prevent oxidative stress. SOD, GPx, and CAT act as antioxidant enzymes that inhibit free radical-related damage and eliminate excessive free radicals in vivo. SOD can catalyze the transformation of superoxide anions into oxygen and hydrogen peroxide, further decreasing the levels of reactive oxygen species (ROS). GPx can catalyze the production of GSH, an antioxidant that interacts with ROS. MDA is a toxic molecule and acts as a biomarker that reflects the level of oxidative damage in the liver. In this work, these markers of oxidative stress in the liver tissue of the model group were essentially changed compared to the control group (*p* < 0.05). CBN treatment alleviated the impairment of oxidative equilibrium (Figure 2). Our results indicated that all three doses of CBN (200–800 mg/kg/BW) effectively restored the abnormal level of MDA and activity of CAT caused by the injection of D-Gal. However, the low dose of CBN (200 mg/kg/BW) hardly ameliorated the decreased activities of SOD and GPx in the model group. This result is similar to those of a previous study by Qian’s group [17], in which 200 mg/kg/BW torularhodin also hardly ameliorated decreased SOD activity in a D-Gal-induced liver injury model.

The amelioration of oxidative stress is also reflected with serum biochemical indices, including ALT and AST. It is critical that oxidative stress affects lipid peroxidation. ALT and AST are fundamentally situated in the hepatocyte cell layer. After D-Gal injection, D-Gal caused liver injury based on the liver histology (Figure 4). An excessive amount of free radicals leads to the lipid peroxidation of cell unsaturated fats. Damage to the construction of the hepatocyte cell film leads to the impairment of its impermeability and causes the release of ALT and AST into the circulatory system. Similar to D-Gal-induced liver injury, an alcoholic liver injury mouse model also exhibited reduced serum levels of GSH and SOD [18]. Repairing the oxidative equilibrium is an effective method for protecting against liver injury. In this work, treatment with different doses of CBN restored the serum biochemical indices (ALT and AST) as well as antioxidative enzymes (SOD, CAT, and GPx). Similar to our work, bioactive compounds from natural resources have been found to exhibit great hepatoprotective effects in a D-Gal-induced liver injury model. Ginsenoside isolated from red ginseng (*Panax ginseng* C.A Meyer) inhibited liver injury caused by injection of D-Gal and decreased the level of ALT and antioxidant activity, such as CAT and SOD [19]. Similarly, saponins identified from *P. ginseng* inhibited marker levels of oxidative stress and inflammatory cytokines induced by D-Gal in serum and liver tissue [20]. Polyphenols extracted from traditional medical herb *Apocynum venetum* also reversed the decreased levels or activities of MDA, SOD, and GPx caused by exposing mice to oxidative stress [21]. Hence, the administration of CBN, similar to the above-reported natural bioactive compounds, ameliorates D-Gal-induced oxidative stress and protects liver hepatocytes from lipid peroxidation.

In addition to oxidative stress, inflammation also plays a vital role in the aging process. In a previous study, a D-Gal-induced liver injury mouse model exhibited an increment in pro-inflammatory cytokine levels, contrasting with the control group [17]. In this work, increased levels in the serum (IL-1β and IL-6) and liver tissue (TNF-α, IL-1β and IL-6) were effectively reduced via treatment with CBN (Figure 1 and Figure 3). After CBN treatment, the serum levels of IL-1β and IL-6 diminished by 28.8% (*p* < 0.05) and 30.5% (*p* < 0.05), respectively. Furthermore, the TNF-α, IL-1β, and IL-6 levels in the liver tissue were reduced when compared with the model group (*p* < 0.05). The anti-inflammatory effects of CBN highlighted in this work are in accordance with previous studies concerning natural bioactive compounds. For example, green-tea polyphenols exhibit inhibitory effects against the increased level of pro-inflammatory cytokines (IL-1β and IL-6) in serum and liver tissue caused by injection of D-Gal [22]. Lycopene, mainly found in tomatoes, effectively attenuates the D-Gal-induced inflammatory response and suppresses oxidative stress [23]. Glucosides and quercetin from *Rosa roxburghii* Tratt fruit significantly decrease the contents of IL-1β, IL-6, and TNF-α in serum and liver tissue [24]. As an isoflavone, puerarin isolated from herb radix *Puerariae* exerts a regulatory effect against the inflammatory response by inhibiting the expression of pro-inflammatory cytokines in D-Gal-induced liver injury mice [25]. In our D-Gal-induced liver injury mouse model, the findings gave similar conclusions and indicated that CBN also exhibits significant immunosuppressive activity. The JAK2/STAT3 signaling pathway is a significant pathway for cytokine signal transduction, and is generally engaged with cytokine overflow. The JAK family contains four members, i.e., JAK1, JAK2, JAK3, and TYK2, which are related to receptors via various patterns. JAK2 activation and phosphorylated JAK2 (P-JAK2) modulate and promote the degree of phosphorylated STAT3 (P-STAT3), which moves from the membrane into the nucleus, and regulate the expressions of target genes [16]. In a rat model of liver injury associated with severe acute pancreatitis, the inhibitor AG490 of JAK2 effectively reduced the serum levels of TNF, IL-6, and IL-18 by blocking excessive JAK2 and STAT3 activation [26]. In an anti-tuberculosis drug-induced liver injury rat model, pyrrolidine dithiocarbamate effectively decreased the serum level of cytokines (TNF-α, IL-1β, and IL-6) by repressing the phosphorylation of JAK2 and STAT3 [27]. P-JAK2 and P-STAT3 levels were significantly lower with CBN treatment (*p* < 0.05).

According to a previous summary [28], JAK2 also regulates MAPK and other downstream signaling pathways. The MAPK family mainly comprises Erk1/2, JNK, and p38. The MAPK protein family is activated via the phosphorylation of these proteins, which further activate transcription factors regulating the expression of target genes in the nucleus. In a previous study, urantide inhibited liver injury in vivo by inhibiting the MAPK signaling pathway [29]. In our study, the model group presented higher levels of phosphorylated JAK2 and phosphorylated p38 (Figure 5), indicating the MAPK signaling pathway’s involvement in D-Gal-induced liver injury in vivo. Western blot analysis revealed that transcription factor NF-κB phosphorylation was increased in the model group in vivo. In a previous study, some transcription factors including NF-κB and STAT3 were connected to liver diseases [30,31]. Yan et al. [32] revealed that the silencing receptor TLR5 can successfully alleviate oxidative stress and inflammation processes by inhibiting the MAPK/NF-κB signaling pathway in hyperammonemia-induced liver injury in vivo. Similar results were obtained in some research on didymin, a flavone compound isolated from the plant *Origanum vulgare* that exhibits hepatoprotective effects in CCl_4_-induced liver injury via the regulation of the MAPK/NF-κB signaling pathway in vivo [33]. Osthole, a coumarin-derivative compound, enhances D-Gal-induced liver injury by inhibiting TLR4 and its downstream MAPK/NF-κB signaling pathway [34]. The MAPK/NF-κB signaling pathway was also found to be involved in acute liver injury in a dog model. Tao et al. [35] announced that extracts from *Penthorum Chinense* Pursh had a hepatoprotective effect against CCl_4_-induced acute liver injury via the MAPK/NF-κB signaling pathway. Hence, CBN exerts its hepatoprotective effect against D-Gal-induced liver injury by inhibiting the phosphorylation of JAK2, which further down-regulates the activation of two transcription factors, STAT3 and NF-κB, and related downstream signaling pathways.

## 4. Methods and Materials

### 4.1. Reagents

**Figure 6:**
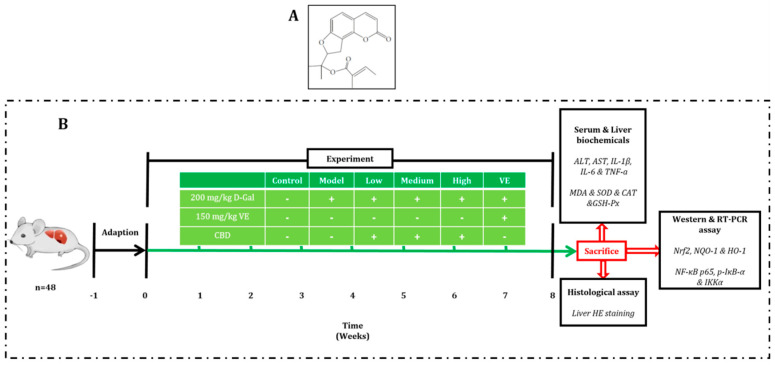
Chemical structure of CBN (**A**) and diagram of the design of the animal experiment (**B**). After acclimatizing for 1 week, mice in all groups were sacrificed, and samples of their blood and liver tissue were collected for analysis.

Standard CBN (98.5% pure, lot no.: J17IA220297) was purchased from Shanghai Yuanye Bio-Technology Co. (Shanghai, China) and its chemical structure is shown in Figure 6A. Corn oil was obtained from Golden Dragon Fish (Taizhou, Zhejiang, China). Commercial kits for superoxide dismutase (SOD), catalase (CAT), malondialdehyde (MDA), urea nitrogen (UN), and alanine aminotransferase (ALT) were purchased from Nanjing Jiancheng Technology Co. (Nanjing, Jiangsu, China). Antibodies for the Western blot analysis were purchased from Cell Signaling Technology (Beverly, MA, USA).

### 4.2. Animal Experiment

For this study, 48 ICR male mice (8 weeks old, male, 20 ± 2 g) [SPF, SYXK (ZHE) 2018-0013] were provided by Hanzhou Ziyuan Biotech. Co. (Zhejiang, China). The mice were subjected to a 12 h light/dark schedule under invariant conditions (23 ± 1 °C and 60% humidity) and were provided with a normal diet of food and water ad libitum for 1 week. All mice (*n* = 48) were randomly assigned to 6 groups (Figure 6B): a control group (injected with 0.9% saline and fed with corn oil), a model group (injected with D-Gal dissolved in saline and fed with corn oil), a VE group (injected with D-Gal dissolved in saline and fed with 150 mg/kg/BW of VE dissolved in corn oil), and three CBN groups (injected with D-Gal dissolved in saline and fed with 200/400/800 mg/kg/BW CBN dissolved in corn oil). The mice in all groups except the control group were intraperitoneally administered 200 mg/kg/BW of D-galactose once a day for 8 weeks. The handling and sacrificing of all animals were performed as per the *Guide for the Care and Use of Laboratory Animals* (The Ministry of Science and Technology of China, 2006) and the study proposal was approved by the Committee of Taizhou University [no. TZXY-2021-20211007].

### 4.3. Determination of Body Weight and Organ Indices

The BWs of the mice were recorded once a week. After the last treatment, all mice were sacrificed via cervical dislocation. Blood samples (300–500 μL) were collected from eyeball blood, and liver organs from different groups were also collected. The serum was obtained via centrifugation at 2000× *g* and 4 °C for 15 min. The livers, spleens, and kidneys were isolated and weighed for organ index calculation. Each organ index (mg/g) was evaluated using the following formula: organ index = organ weight (mg)/body weight (g).

### 4.4. Determination of Biochemical Indices in Serum and Liver

The serum levels of ALT, AST, IL-1β, and IL-6 and the activities of SOD, CAT, MDA, and GPx in the liver were determined using assay kits (Nanjing Jiancheng Bioengineering Institute, Nanjing, China) following the manufacturer’s instructions. Briefly, the serum and liver tissue samples were diluted approximately 10–50 times and then incubated at 30 °C with various antibodies in the kits. The levels or activities of different proteins were calculated according to standard curves, which used relevant pure standard proteins provided by the manufacturer.

To detect the liver indices, the collected tissue was cut into 1 × 1 mm pieces, and then mixed with an amount of pre-chilled 1.15% potassium chloride solution that was 10 times the volume of the tissue. After homogenization, the obtained homogenates were centrifuged (2000× *g*, 4 °C for 10 min) and the supernatants were used for analysis. Protein concentrations of the liver homogenates were measured with a total protein assay kit (Nanjing Jiancheng Bioengineering Institute, Nanjing, China) and bovine serum albumin was used as the standard.

### 4.5. Tissue Preparation and Histological Analysis

Freshly collected mouse liver organs from the 6 groups were fixed with 4% paraformaldehyde solution at room temperature for 48 h, embedded in paraffin, and sliced into 5 μm sections for routine hematoxylin and eosin (H&E) staining. Histopathological changes in the organs were determined using a microscope at 400 magnification. The Suzuki Score System was used to examine the histopathological evaluation of liver tissue by an experienced pathologist.

### 4.6. Western Blot Analysis

The mouse livers stored at −80 °C from various groups were weighed and mixed with RIPA buffer containing a protease inhibitor cocktail to make a homogenate. The formed solution was centrifuged and the total protein contents were determined using a total protein assay kit (Nanjing Jiancheng Bioengineering Institute, Nanjing, China). The proteins in the lysates were separated using 10% sodium dodecyl sulfate–polyacrylamide gel electrophoresis (SDS-PAGE) and then transferred to polyvinylidene difluoride (PVDF) membranes. The membranes were blocked with 5% skimmed milk and sequentially treated with a primary antibody and horseradish peroxidase-conjugated antibodies.

### 4.7. Statistical Analyses

Data were expressed as the mean ± SD (n = 8). Statistical analyses were performed suing SPSS 20.0 software (SPSS Inc., Chicago, IL, USA). A one-way ANOVA was performed followed by a post hoc test. Differences with *p*-values of <0.05 were considered statistically significant.

## 5. Conclusions

In summary, as manifested by the serum changes and pathological reduction in liver histology, our study provides solid evidence that CBN exhibits great hepatoprotective and inflammatory effects in D-Gal-induced liver injury mice in vivo. Mechanistic studies demonstrated that the protective effect of CBN treatment against liver injury is closely related to the JAK2/STAT3 and JAK2/p38/NF-κB signaling pathways. These data support the conclusion that CBN may be an alternative candidate to use as an immunosuppressive agent and bioactive ingredient for functional food development.

## Figures and Tables

**Table 1:** Effect of columbianetin on body weights and liver indices in mice with D-galactose-induced liver injury.

Groups	Weight (g)		Liver Indices (mg/g)	Kidney Indices (mg/g)
	Initial	Final		
Control	31.07 ± 0.68	47.17 ± 3.79 ^d^	17.01 ± 2.34 ^d^	5.77 ± 0.67
Model	29.98 ± 0.91	33.75 ± 1.19 ^a^	10.61 ± 0.48 ^a^	4.94 ± 0.98
CBN (200 mg/kg)	30.62 ± 1.24	35.88 ± 2.93 ^b^	11.21 ± 1.77 ^b^	4.74 ± 0.68
CBN (400 mg/kg)	30.11 ± 1.62	35.50 ± 1.29 ^b^	11.73 ± 0.98 ^b^	4.48 ± 0.43
CBN (800 mg/kg)	29.54 ± 0.53	35.38 ± 2.75 ^b^	12.37 ± 0.82 ^c^	5.01 ± 0.38
Vitamin E (150 mg/kg)	30.67 ± 0.78	37.33 ± 3.40 ^c^	12.63 ± 0.30 ^c^	5.11 ± 0.79

Different letters ^a,b,c,d^ indicate significant differences (*p* < 0.05).

## Data Availability

Data is contained within the article.

## Abbreviations

ALT	Alanine aminotransferase	JNK	c-Jun N-terminal kinase
APR	*Angelicae pubescentis* radix	MAPK	Mitogen-activated protein kinase
AST	Aspartate aminotransferase	NF-κB	Nuclear factor kappa-light-chain-enhancer of activated B cells
BW	Body weight	OE	Osthole
CAT	Catalase	p38	Protein 38
CBA	Columbianetin acetate	p65	Protein 65
CBN	Columbianadin	P-p38	Phosphorylated protein 38
CBT	Columbianetin	P-p65	Phosphorylated protein 65
D-Gal	D-galactose	P-JAK	Phosphorylated Janus kinase
ELISA	Enzyme-linked immunosorbent assay	P-STAT	Phosphorylated signal transducer and activator of transcription
Erk	Extracellular signal-regulated protein kinase	PVDF	Polyvinylidene difluoride
GSH	Glutathione	ROS	Reactive oxygen species
GPx	Glutathione peroxidase	SDS-PAGE	Sulfate–polyacrylamide gel electrophoresis
H&E	Hematoxylin and eosin	SOD	Superoxide dismutase
ICR	Institute of Cancer Research	STAT	Signal transducer and activator of transcription
IL-1	Interleukin-1	TLR	Toll-like receptor
IL-6	Interleukin-6	TNF-α	Tumor necrosis factor-α
JAK	Janus kinase	VE	Vitamin E
